# Stent placement combined with intraluminal radiofrequency ablation and hepatic arterial infusion chemotherapy for advanced biliary tract cancers with biliary obstruction: a multicentre, retrospective, controlled study

**DOI:** 10.1007/s00330-021-07716-0

**Published:** 2021-02-13

**Authors:** Qing Gou, Lingeng Wu, Wei Cui, Zhiqiang Mo, Dejin Zeng, Liming Gan, Jian He, Qicong Mai, Feng Shi, Meng Chen, Zhonghai Sun, Yongdong Liu, Jingjing Wu, Xiumei Chen, Wenhang Zhuang, Rongde Xu, Weike Li, Qichun Cai, Jing Zhang, Xiaoming Chen, Jiaping Li, Zejian Zhou

**Affiliations:** 1 Department of Interventional Therapy, Cancer Center, Guangdong Provincial People’s Hospital, Guangdong Academy of Medical Sciences, School of Medicine, South China University of Technology, 106 Zhongshan Second Road, Guangzhou, 510080 Guangdong China; 2 Department of Gastroenterology, Zhongshan Hospital of traditional Chinese Medicine, Zhongshan, 528400 Guangdong China; 3 Department of General Surgery, Guangdong Provincial Peoples Hospital, Guangdong Academy of Medical Sciences, Guangzhou, 510080 Guangdong China; 4 Department of Catheterization Lab, Guangdong Provincial People’s Hospital, Guangdong Academy of Medical Sciences, Guangzhou, 510080 Guangdong China; 5 Cancer Center, Guangdong Provincial People’s Hospital, Guangdong Academy of Medical Sciences, School of Medicine, South China University of Technology, Guangzhou, 510080 Guangdong China; 6 Department of Oncology, Cancer Center, Guangdong Clifford Hospital, Guangzhou, 511400 Guangdong China; 7 Department of Interventional Oncology, the First Affiliated Hospital, Sun Yat-sen University, Guangzhou, 510080 Guangdong China

**Keywords:** Catheter ablation, Infusions, Intra-arterial, Biliary tract neoplasms, Cholestasis

## Abstract

**Objective:**

To evaluate the efficacy and safety of stent placement combined with intraluminal radiofrequency ablation (intra-RFA) and hepatic arterial infusion chemotherapy (HAIC) for patients with advanced biliary tract cancers (Ad-BTCs) and biliary obstruction (BO).

**Methods:**

We retrospectively reviewed data for patients with Ad-BTCs and BO who underwent stent placement with or without intra-RFA and HAIC in three centres between November 2013 and November 2018. The stent patency time (SPT), overall survival (OS), and adverse events (AEs) were analysed.

**Results:**

Of the 135 enrolled patients, 64 underwent stent placement combined with intra-RFA and HAIC, while 71 underwent only stent placement. The median SPT was significantly longer in the combination group (8.2 months, 95% confidence interval [CI]: 7.1–9.3) than in the control group (4.3 months, 95% CI: 3.6–5.0; *p* < 0.001). A similar result was observed for OS (combination: 13.2 months, 95% CI: 11.1–16.5; control: 8.5 months, 95% CI: 7.6–9.6; *p* < 0.001). The incidence of AEs related to biliary tract operation was not significantly different between the two groups (*p* > 0.05). The most common AE and serious AE related to HAIC were alanine aminotransferase elevation (24/64; 37.5%) and thrombocytopenia (8/64; 12.5%), respectively. All AEs were tolerable, and there was no death from AEs.

**Conclusions:**

Stent placement combined with intra-RFA and HAIC may be a safe, potential treatment strategy for patients with Ad-BTCs and BO.

**Key Points:**

*• Advanced biliary cancers (Ad-BTCs) with biliary obstruction (BO) can rapidly result in liver failure and cachexia with an extremely poor prognosis.*

*• Stent placement combined with intraluminal radiofrequency ablation and hepatic arterial infusion chemotherapy may be safe and effective for patients with Ad-BTCs and BO.*

*• The long-term efficacy and safety of the combined treatment is promising.*

## Introduction

Biliary tract cancers (BTCs) are classified into intrahepatic cholangiocarcinoma, perihilar cholangiocarcinoma (pCCA), distal cholangiocarcinoma, gall bladder cancer, and carcinoma of the ampulla according to the anatomical structure [1, 2]. The incidence and mortality of BTCs have progressively increased worldwide in recent decades [3, 4]. For early-stage disease, liver transplantation and radical resection seem to be potential curative options [5]. Unfortunately, approximately 60–70% of patients are diagnosed in advanced stages, and > 50% of advanced BTCs (Ad-BTCs) are complicated by biliary obstruction (BO) [6], which can rapidly result in liver failure and cachexia with an extremely poor prognosis [7]. The median survival of patients with Ad-BTCs and BO is 4.8 months, with a 5-year survival rate of 10% [8]. Metal stent placement is considered the most effective palliative treatment to improve the quality of life and survival [9]. However, 1-year recurrence rates for BO reach 68%, with the main cause for recurrence being local tumour progression [10, 11]. Therefore, tumour growth control is critical for maintaining stent patency and prolonging survival in cases of Ad-BTCs with BO.

Radiofrequency ablation (RFA) is widely used to treat solid tumours, particularly hepatocellular carcinoma [12]. Intraluminal RFA (intra-RFA), based on the same principle of energy generation, has been used to manage malignant BO (MBO) in recent years [13]. The high thermal energy generated by radiofrequency electrodes can effectively hamper intraluminal tumour growth [14]. It was reported that intra-RFA could prolong stent patency and survival in patients with BTCs and BO, although its efficacy needs improvement [15]. In a meta-analysis, the pooled weighted mean difference in stent patency was only 1.7 months [16]. Moreover, intra-RFA cannot reach residual tumours and lesions located outside the biliary tract, which may quickly progress and trigger stent re-stenosis.

Hepatic arterial infusion chemotherapy (HAIC) can effectively increase the local concentration of anti-tumour drugs and decrease the systemic distribution of agents [17]. Compared with systemic chemotherapy, HAIC can significantly reduce adverse events (AEs) and achieve more potent local anti-tumour effects [18]. Patients with advanced hepatocellular carcinoma can achieve high local tumour control rates and yield long-term benefits from HAIC [19, 20], and retrospective studies suggest that HAIC can also offer clinical benefits to patients with Ad-BTCs [21, 22]. A recent phase 2 clinical study indicated that HAIC with oxaliplatin and 5-fluorouracil for advanced pCCA can effectively control local tumour growth and provide an encouraging survival benefit [23]. Tumours that cannot be completely ablated and lesions outside the biliary tract can be simultaneously controlled by compensating HAIC. Intra-RFA combined with HAIC with oxaliplatin and 5-fluorouracil may be a promising strategy for Ad-BTCs with BO. To our knowledge, there is no report regarding this new treatment modality. Therefore, we conducted a multicentre, retrospective study to evaluate the efficacy and safety of stent placement + intra-RFA+HAIC for Ad-BTCs with BO.

## Materials and methods

### Study design and patients

This retrospective, multicentre cohort study was approved by the Ethics Committees of Guangdong Provincial People’s Hospital, Zhongshan Hospital of Traditional Chinese Medicine, and Guangdong Clifford Hospital, and the requirement for written informed consent was waived for all patients.

**Fig. 1:**
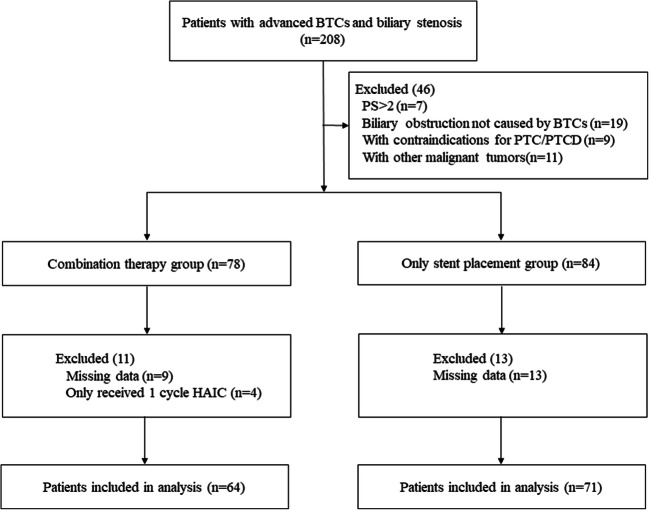
Flow diagram of patient selection

We retrospectively reviewed the medical records of consecutive patients who received interventional treatments for Ad-BTCs with BO in the three aforementioned centres between November 2013 and November 2018 (Fig. 1). According to the eighth edition of the American Joint Committee on Cancer TNM staging system, stage III and stage IV BTCs were defined as Ad-BTCs [24]. Patients who received stent placement + intra-RFA+HAIC with oxaliplatin and 5-fluorouracil were included in the combination group, while patients who underwent stent placement only were included in the control group. In the combination group, patients underwent intra-RFA and stent placement simultaneously with percutaneous transhepatic cholangiography (PTC) or after percutaneous transhepatic cholangial drainage (PTCD), followed by HAIC when Child–Pugh class A or B liver function was achieved. Patients in the control group only underwent stent placement simultaneously with PTC or after PTCD. The stent patency time (SPT) and overall survival (OS) were the primary endpoints, while treatment-related AE rates were secondary endpoints. Relevant clinical data were collected from the medical record system and follow-up records. The follow-up deadline was November 2019.

The inclusion criteria were as follows: (1) age of 18 to 80 years; (2) diagnosis of Ad-BTCs through pathology or cytology combined with MRCP, PET, or CT; (3) diagnosis of BO by serum bilirubin measurement and CT, MRCP, or PET; and (4) patient’s life expectancy > 1 month. The exclusion criteria were as follows: (1) Eastern Cooperative Oncology Group performance status > 2, (2) BO not associated with BTC, (3) other malignancies, (4) contraindications for PTC/PTCD, (5) only one HAIC cycle, and (6) missing data.

### Intra-RFA and stent placement

**Fig. 2:**
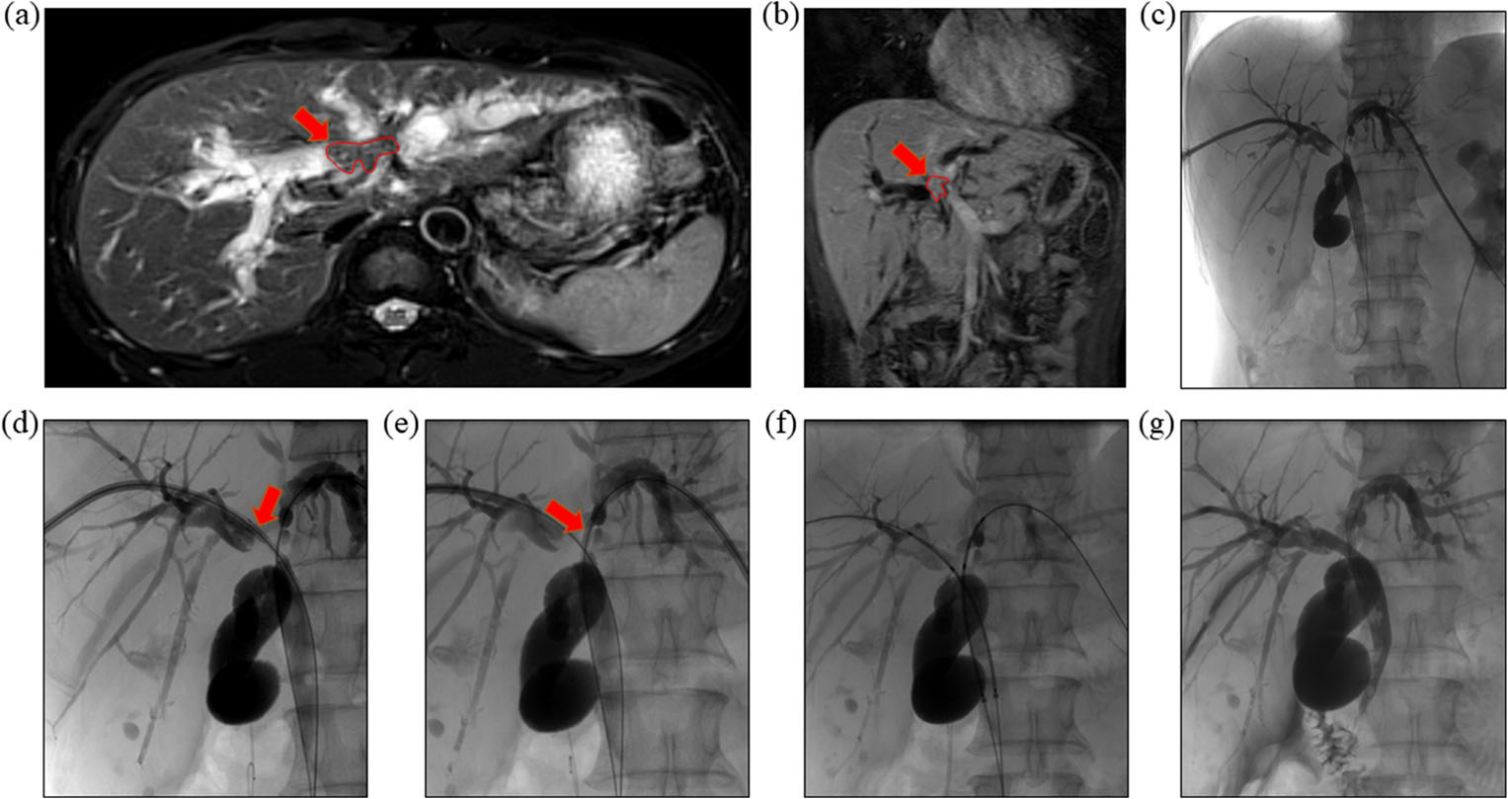
Intra Intraluminal radiofrequency ablation and double stent-by-stent deployment. **a**, **b** MRCP and contrast-enhanced CT showed perihilar cholangiocarcinoma presented malignant obstructive jaundice. **c–e** By adding a hard-exchange guide wire and sending into the ablation catheter, radiofrequency ablation (red arrow) was performed on the left and right bile duct stenosis segment. **f**, **g** After balloon expansion, the uncovered metal stent was implanted, and angiography showed that the stent was expanded well, and the common bile duct was restored unobstructed

After successful puncture of intrahepatic bile ducts using percutaneous access set (NPAS-100-RH-NT; Cook Medical) (Fig. 2c), a catheter (NHB5.0-38-40-P-NS-KMP; Cook Medical) was introduced into the biliary system using a guidewire. The site and length of the obstruction were measured according to the cholangiography findings. If the guidewire failed to pass through the obstructed segment, PTCD was performed.

In the combination group, intra-RFA was performed before stent placement. In order to prevent biliary tract infections, all patients received prophylactic antibiotics the day before intra-RFA. An ablation catheter (Habib EndoHPB; EMcision Ltd.) was inserted into the targeted section under the guidewire passed through the obstructed segment. Subsequently, the ablation catheter probe was connected to a frequency generator (RITA 1500X; AngioDynamics), and ablation was performed with a power of 10 W for 120 s (Fig. 2d, e). If the stenosis length was > 2.0 cm, step-by-step intra-RFA was performed from the distal to the proximal end. For patients with multiple intrahepatic stenoses, the bile ducts simultaneously underwent ablation do not exceed 3 ducts. Subsequently, an uncovered metal stent (Wallstent; Boston Scientific) (Fig. 2f, g) with an 8 mm diameter was placed. In the control group, patients underwent stent placement using the same technique. In both groups, a multi-purpose drainage catheter was placed in the bile duct for 3–5 days after stent placement, and it was removed once stent patency was confirmed by cholangiography.

### HAIC

A 5-French catheter (HNB5.0-38-80-P-NS-RH; Cook Medical) was inserted into the coeliac trunk or superior mesenteric artery. According to arteriography results, the catheter was selectively placed in the main feeding arteries of the tumours. To prevent obstruction by a blood clot, the end of the catheter was locked with a heparin lock (10 ml, 10,000 units, 1:1000 dilution). The peripheral part and vascular sheath were properly fixed on the groin, and the catheter tip position at the target location was confirmed by fluoroscopy. Subsequently, HAIC was initiated in the general ward using the following regimen: oxaliplatin, 130 mg/m^2^ from hour 3 on day 1; leucovorin, 400 mg/m^2^ from hour 2 on day 1; and fluorouracil, 400 mg/m^2^ bolus at hour 3 and 2400 mg/m^2^ over 46 h on days 1 and 2. For the alleviation of AEs, dexamethasone, stomach-protecting agents, and antiemetic drugs were administered to patients 30 min before HAIC. The same procedures were repeated for the next HAIC cycle. Each cycle was 3 weeks, and a total of six cycles were required. Delays and modifications were allowed for HAIC-related AEs. HAIC was discontinued in case of BO recurrence, unacceptable toxic effects, patient refusal to continue, or change to other treatments.

### Assessments and follow-up

SPT was calculated from the date of stent placement to the date of BO recurrence confirmed by imaging examination. If patients were lost to follow-up, SPT was calculated from the date of stent placement to the date of the last follow-up and considered censored data. If there was no concrete evidence of BO recurrence during the patient’s lifespan, SPT was considered equivalent to OS and considered censored data. OS was calculated from the date of stent placement to the date of death or the date of the last follow-up. Treatment-related complications were defined as AEs within 2 weeks after intra-RFA, stent placement, or HAIC. AE grades were determined using the CTCAE5.0 criteria; grade 1–2 AEs were considered mild-to-moderate AEs while grade 3–4 AEs were considered serious AEs (SAEs).

In the first month, laboratory tests were completed within 1–2 weeks after stent placement in all patients. From the second month, laboratory tests were performed at intervals of 3 weeks to 2 months. If serum bilirubin levels were increased (> 5 mg/dL), CT or MRI was performed to confirm BO recurrence. To evaluate HAIC-related AEs, laboratory tests were performed every 1–2 weeks after HAIC. In cases of HAIC treatment withdrawal, patients were contacted every 2–3 months until they died or were lost to follow-up.

### Statistical analysis

The independent-sample *t* test, the chi-squared test, and Fisher’s exact test were used, as appropriate, to determine differences in clinical variables, the clinical efficacy, and AE rates between the two groups. SPT and OS were calculated using the Kaplan–Meier method, and differences were identified using the log-rank test. Statistically significant factors (including clinical and laboratory parameters and tumour characteristics predicting SPT and OS) in univariate analysis were included in multivariate Cox regression analysis. A Cox proportional hazards model was used to estimate the hazard ratios. A *p* value of < 0.05 was considered statistically significant. All statistical analyses were performed using Statistical Package for SPSS software, version 25.0 (IBM) and STATA version 16.0 (Stata Corporation).

## Results

### Patient characteristics

In total, 206 consecutive patients with Ad-BTCs and BO underwent interventional treatments during the study period. After application of the inclusion and exclusion criteria, 135 (82 men [median age, 62.0 years; range, 35.0–84.0 years] and 53 women [median age, 61.0 years; range, 34–82 years]) were enrolled (Fig. 1). There were 64 and 71 patients in the combination and control groups, respectively, with no significant between-group differences in baseline characteristics (Table 1).

**Table 1:** Baseline clinical characteristics of patients in the two groups

	Combination group (*n* = 64)	Control group (*n* = 71)	*p*
Gender			0.270
Male	42 (65.6)	40 (56.3)	
Female	22 (34.4)	31 (43.7)	
Age a (year)	60.0 ± 12.7	62.0 ± 12.6	0.848
≥ 65	26 (40.6)	30 (42.3)	
< 65	38 (59.4)	41 (57.7)	
ECOG performance status +			0.176
1	45 (70.3)	42 (59.2)	
2	19 (29.7)	29 (40.8)	
Primary site of tumour			0.932
Intrahepatic cholangiocarcinoma	7 (10.9)	8 (11.3)	
Perihilar cholangiocarcinoma	18 (28.1)	17 (23.9)	
Extrahepatic cholangiocarcinoma	18 (28.1)	25 (35.2)	
Gallbladder carcinoma	7 (10.9)	7 (9.9)	
Carcinoma of ampulla	14 (21.9)	14 (19.7)	
Level of biliary obstruction ‡			0.792
Common bile or hepatic duct (type I)	50 (78.1)	53 (74.6)	
Hepatic confluence			
Type II	0	1 (1.4)	
Type III (a/b)	13 (20.3)	14 (19.7)	
Intrahepatic (type IV)	1 (1.6)	3 (4.2)	
TNM stage			0.247
III	19 (29.7)	30 (42.3)	
IV	43 (67.2)	38 (53.5)	
Distant metastasis			0.117
Yes	23 (35.9)	35 (49.3)	
No	41 (64.1)	36 (50.7)	
Obstructive length a(cm)	3.9 ± 1.6	3.8 ± 1.1	0.375
≥ 4	31 (48.4)	29 (40.8)	
< 4	33 (51.6)	42 (59.2)	
Previous treatment			
Surgical resection	10 (15.6)	9 (12.7)	0.623
Radiotherapy	4 (6.3)	3 (4.2)	0.888
Adjuvant chemotherapy	1 (1.6)	5 (7.0)	0.261
Preoperative total bilirubin a(μ mol/L)	291.7 ± 129.6	297.3 ± 118.4	0.850
CA125 (U/mL)			0.359
> 40	31 (48.4)	40 (56.3)	
≤ 40	33 (51.6)	31 (43.7)	
CA19-9 (U/mL)			0.582
> 40	57 (89.1)	61 (85.9)	
≤ 40	7 (10.9)	10 (14.1)	
Infected markers			
WBC (×10^9^/L )			0.227
> 8	34 (53.1)	45 (63.4)	
≤ 8	30 (46.9)	26 (36.6)	

^a^Data are medians ± standard deviation

^+^According to the Toxicity and response criteria of the Eastern Cooperative Oncology Group

^‡^According to the Bismuth classification of perihilar cholangiocarcinoma

Unless otherwise indicated, data are number of patients and data in parentheses are percentages

*HAIC*, hepatic artery infusion chemotherapy; *CA125/19-9*, carbohydrate antigen 125/19-9; *WBC*, white blood cell count

### Intervention

All enrolled patients received stents that were placed simultaneously with PTC in 78 patients (57.8%) and after PTCD in 57 patients (42.2%). The stent length was > 6 cm and ≤ 6 cm in 48 (34.1%) and 87 (64.5%) patients, respectively. Post-stenting bilirubin levels at 1 and 2 weeks were significantly reduced in both groups, with no significant between-group difference (*p* > 0.05). In the combination group, all patients subsequently underwent successful intra-RFA, followed by successful insertion of the arterial catheter and administration of at least two HAIC cycles. A median number of 4.64 HAIC cycles (2–6) per patient was recorded for the overall cohort. All operations were performed under local anaesthesia.

### Stent patency time and overall survival

**Fig. 3:**
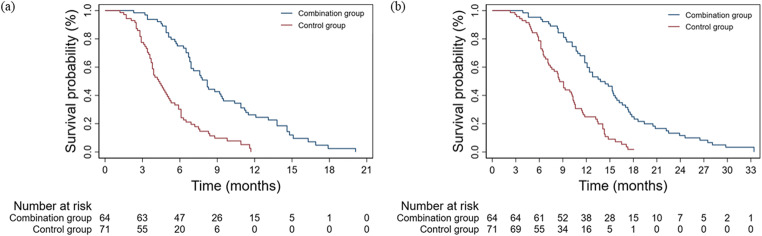
Stent patency and overall survival. **a** Cumulative stent patency by the Kaplan–Meier analysis. Median stent patency was 7.8 months in the combination therapy group versus 4.0 months in the only stent group. **b** Significantly better overall survival rates were observed in the patients treated with combination therapy according to the Kaplan–Meier method (*p* < 0.001 for both)

The median follow-up duration was 10.6 (range: 7.2–14.8) months, and the longest duration was 33.4 months. Ten patients (7.4%) were alive at the last follow-up. BO recurred in 123 patients (91.1%). The median SPT was significantly longer in the combination group (8.2 months, 95% confidence interval [CI]: 7.1–9.3) than in the control group (4.3 months, 95% CI: 3.6–5.0; *p* < 0.001; Fig. 3a). The median OS showed a similar result (combination: 13.2 months, 95% CI: 11.1–16.5; control: 8.5 months, 95% CI: 7.6–9.6; *p* < 0.001; Fig. 3b). The 3-month survival rate (100.0% vs. 97.2%) was comparable between groups (*p* = 0.523). However, the 6-, 9-, and 12-month survival rates were significantly higher in the combination group (95.3% vs. 77.5%, 81.3% vs. 47.9%, 59.4% vs. 22.5%; *p* < 0.05).

**Fig. 4:**
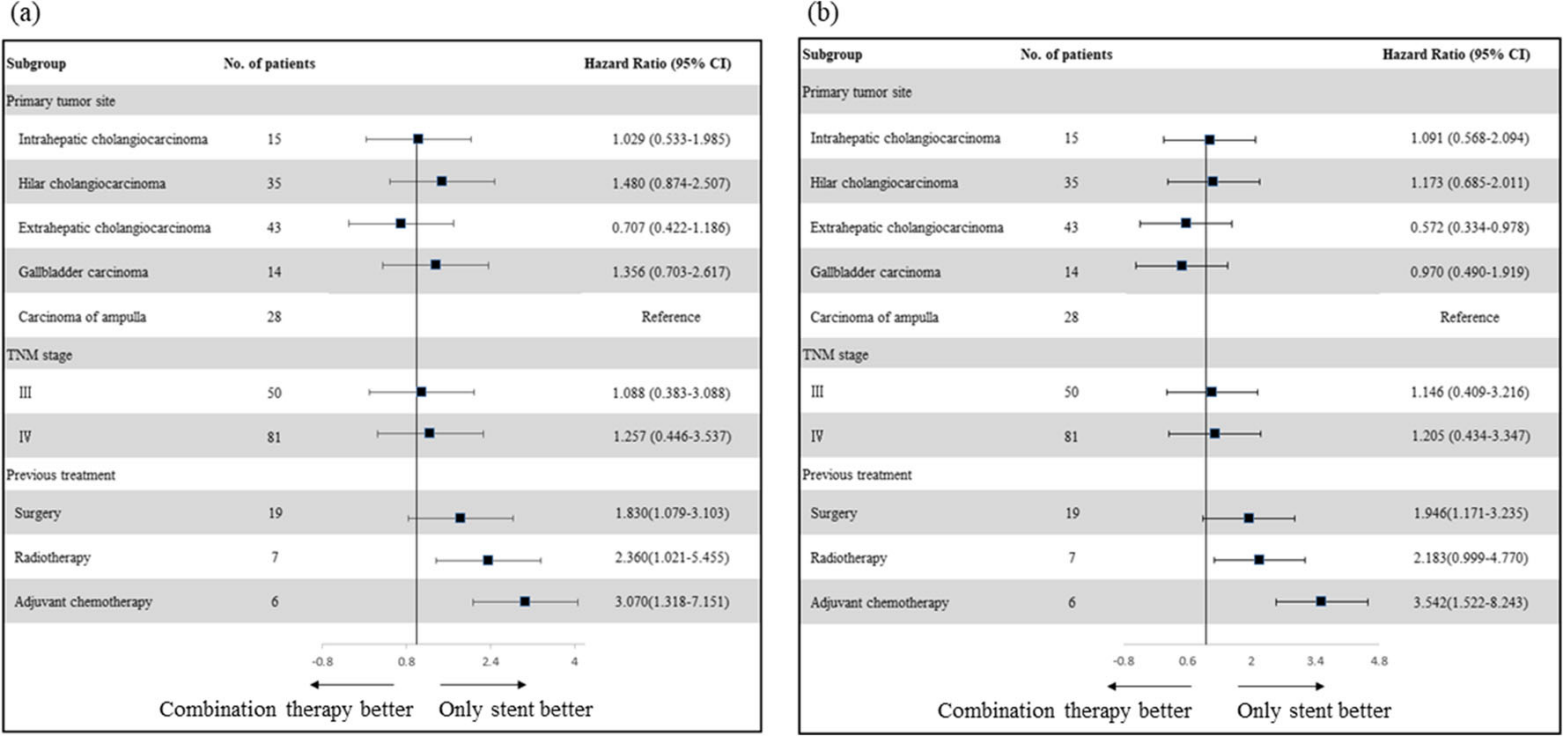
**a** Forest plot of OS and (**b**) stent patency according to prespecified subgroups. HR, hazard ratio

Univariate and multivariate Cox regression analyses for factors relevant to SPT and OS are shown in Tables 2 and 3. The absence of distant metastasis, a shorter stricture (< 4 cm), and the level of BO in Bismuth types I–II were independent predictors of longer SPT and OS. These factors remained significant in multivariate analysis. Figure 4 presents the hazard ratios for OS (a) and SPT (b) according to the potential influencing baseline factors; a history of previous treatments was associated with shorter SPT and OS.

**Table 2:** Univariate and multivariate analysis of prognostic factors of stent patency in 135 patients with malignant biliary obstruction

			Univariate analysis		Multivariate analysis	
Variable*		No. of cases	Median stent patency (95%CI)*	*p*^†^	HR (95%CI)	*p*^$^
Gender	Male	82	6.1 (5.4–6.8)	0.941		
	Female	53	6.3 (4.1–8.5)			
Age (year)	≥ 65	56	5.6 (4.3–6.9)	0.672		
	< 65	79	6.5 (5.6–7.4)			
EGCO PS +	≥ 2	48	5.6 (4.4–6.8)	0.193		
	< 2	87	6.5 (5.5–7.5)			
Bismuth type ‡	III–IV	31	4.3 (3.2–5.4)	0.000	2.778 (1.765, 4.371)	0.000
	I–II	104	6.8 (6.1–7.5)			
Distant metastasis	Yes	58	5.7 (4.8–6.6)	0.008	1.708 (1.171, 2.491)	0.005
	No	77	6.7 (5.9–7.5)			
CA 125 (U/mL)	> 40	71	5.8 (4.4–7.2)	0.263		
	≤ 40	64	6.5 (5.8–7.2)			
CA 19-9 (U/mL)	> 40	118	6.3 (5.4–7.2)	0.222		
	≤ 40	17	5.9 (4.4–7.4)			
Length of stricture (cm)	≥ 4	60	5.2 (4.2–6.2)	0.027	1.727 (1.124, 2.654)	0.013
	< 4	75	6.8 (6.0–7.6)			
Length of stent (mm)	> 6	48	5.8 (4.3–7.3)	0.646		
	≤ 6	87	6.1 (5.3–6.9)			
Stent placement	Following	57	6.1 (4.4–7.8)	0.661		
	Synchronising	78	6.1 (5.2–7.0)			

*Stent patency data are in months, and the Kaplan–Meier method was used

^†^The long-rank test was used

^$^Cox regression was used

^+^According to the Toxicity and response criteria of Eastern Cooperative Oncology Group

^‡^According to the Bismuth classification of perihilar cholangiocarcinoma

*CA125/19-9*, carbohydrate antigen 125/19-9

**Table 3:** Univariate and multivariate analysis of prognostic factors of OS in 135 patients with malignant biliary obstruction

			Univariate analysis		Multivariate analysis	
Variable*		No. of cases	Median OS (95%CI)*	*p*^†^	HR (95%CI)	*p*^$^
Gender	Male	82	10.5 (9.4–11.6)	0.789		
	Female	53	11.6 (9.9–13.3)			
Age (year)	≥ 65	56	10.6 (9.1–12.1)	0.973		
	< 65	79	11.4 (9.9–12.9)			
EGCO PS +	≥ 2	48	9.9 (7.7–12.1)	0.159		
	< 2	87	11.8 (10.1–13.5)			
Bismuth type ‡	III–IV	31	7.6 (6.0–9.2)	0.000	2.778 (1.765, 4.371)	0.000
	I–II	104	12.1 (10.3–13.9)			
Distant metastasis	Yes	58	10.3 (9.2–11.4)	0.021	1.708 (1.171, 2.491)	0.005
	No	77	11.8 (10.5–13.1)			
CA 125 (U/mL)	> 40	71	10.6 (9.0–12.2)	0.976		
	≤ 40	64	11.4 (10.1–12.7)			
CA 19-9 (U/mL)	> 40	118	10.9 (9.9–11.9)	0.190		
	≤ 40	17	9.1 (4.4–13.8)			
Length of stricture (cm)	≥ 4	60	9.1 (8.0–10.3)	0.000	1.727 (1.124, 2.654)	0.013
	< 4	75	13.4 (11.1–15.7)			
Length of stent (mm)	> 6	48	10.2 (8.2–12.2)	0.379		
	≤ 6	87	11.6 (9.9–13.3)			
Stent placement	Following	57	10.9 (9.5–12.3)	0.365		
	Synchronising	78	10.7 (9.2–12.2)			

*OS data are in months, and the Kaplan–Meier method was used

^†^The long-rank test was used

^$^Cox regression was used

^+^According to the Toxicity and response criteria of the Eastern Cooperative Oncology Group

^‡^According to the Bismuth classification of perihilar cholangiocarcinoma

*OS*, overall survival; *CA125/19-9*, carbohydrate antigen 125/19-9

### Adverse events

AEs related to the percutaneous biliary tract operation procedures are summarised in Table 4. The overall AE incidence was similar in the combination and control groups (mild-to-moderate AEs: 56.3% vs. 53.5%, SAEs: 10.9% vs. 7.0%; *p *= 0.598). The most frequent mild-to-moderate AE and SAE were abdominal pain (21.9% and 22.5% in the combination and control groups, respectively) and biliary infection (4.7% and 2.8% in the combination and control groups, respectively), respectively. The incidences and grades of abdominal pain, haemorrhage, pancreatitis, peritonitis, bile duct perforation, and biliary infection showed no significant between-group differences (*p* > 0.05). There were no AE-related deaths during the follow-up.

**Table 4:** Comparison of post-procedure AEs of biliary tract operation between the two groups

	Combination group (*n* = 64)	Control group (*n* = 71)	*p*
Total AEs			0.598
Grades 1–2 Grades 3–4	36 (56.3) 7 (10.9)	38 (53.5) 5 (7.0)	
Abdominal pain			0.629
Grades 1–2 Grades 3–4	14 (21.9) 0 (0.0)	16 (22.5) 1 (1.4)	
Hemorrhage			0.335
Grades 1–2 Grades 3–4	8 (13.1) 1 (1.6)	6 (8.1) 0 (0.0)	
Pancreatitis			0.780
Grades 1–2 Grades 3–4	5 (7.8) 2 (3.1)	5 (7.0) 1 (1.4)	
Peritonitis			0.480
Grades 1–2 Grades 3–4	3 (4.7) 1 (1.6)	2 (2.8) 0 (0.0)	
Bile duct perforation			0.366
Grades 1–2 Grades 3–4	1 (1.6) 0 (0.0)	0 (0.0) 1 (1.4)	
Biliary infection			0.571
Grades 1–2 Grades 3–4	5 (7.8) 3 (4.7)	9 (12.7) 2 (2.8)	

Data are number of patients and data in parentheses are percentages

*AEs*, adverse events; *HAIC*, hepatic artery infusion chemotherapy

HAIC-related AEs are listed in Table 5. Arterial catheter-related AEs included catheter dislocation, catheter obstruction, arterial thrombosis, arterial haematoma, and catheter infection, observed in three (4.7%), two (3.1%), one (1.6%), five (7.8%), and one (1.6%) patient, respectively. Liver function and haematopoietic function were transiently affected by HAIC. The most common AE was alanine aminotransferase (ALT) elevation (*n *= 31; 48.4%). The most common mild-to-moderate AE and SAE were ALT elevation and thrombocytopenia, which occurred in 24 (37.5%) and eight (12.5%) patients, respectively.

**Table 5:** Treatment-related AEs in the patients treated with HAIC

	Grades 1–2, no. (%)	Grades 3–4, no. (%)
Any	54 (84.4)	8 (12.5)
Arterial cannula related		
Catheter dislocation	3 (4.7)	0
Catheter obstruction	2 (3.1)	0
Arterial thrombosis	1 (1.6)	0
Arterial haematoma	5 (7.8)	0
Catheter infection	1 (1.6)	0
Blood/bone marrow suppression		
Leukocytopenia	9 (14.1)	4 (6.3)
Neutropenia	11 (17.2)	3 (4.7)
Thrombocytopenia	18 (28.1)	8 (12.5)
Hepatic function		
Hyperbilirubinemia	12 (18.8)	5 (7.8)
AST increase	12 (18.8)	6 (9.4)
ALT increase	24 (37.5)	7 (10.9)
GI events		
Abdominal pain	8 (12.5)	1 (1.6)
Anorexia	14 (21.9)	4 (6.3)
Vomiting	19 (29.7)	2 (3.1)
Diarrhea	9 (14.1)	4 (6.3)
Constitutional symptoms		
Fatigue	17 (26.6)	0
Fever	6 (9.4)	0

*AEs*, adverse event; *HAIC*, hepatic artery infusion chemotherapy; *AST*, aspartate transaminase; *ALT*, alanine transaminase

## Discussion

In the present study of patients with Ad-BTCs and BO, we found that SPT and OS were markedly longer in patients treated by stent placement + intra-RFA+HAIC than in those treated by stent placement alone. The absence of metastasis, a stricture of < 4 cm, and the level of BO in Bismuth types I–II were independent predictors of longer SPT and OS. All AEs could be tolerated or relieved after active symptomatic treatments.

In cases of BO caused by BTCs or other malignancies, relieving obstructive jaundice and maintaining stent patency are extremely pivotal to prolong survival and improve the quality of life. In our study, SPT and OS were significantly longer in the combination group than in the control group, with significantly higher survival rates at 6, 9, and 12 months in the former group. These encouraging outcomes can be attributed to the use of intra-RFA+HAIC for restricting local disease progression. The ablation electrode can generate high thermal energy, which induces coagulative necrosis of tumour cells in the bile duct cavity. Monga confirmed the disappearance of tumour blood vessels and lumen enlargement after intra-RFA [25]. Several studies have reported the clinical efficacy, safety, and survival benefit of intra-RFA for MBO [13, 15, 26]. In a prospective, randomised trial comparing intra-RFA + stent placement with stent placement alone for unresectable extrahepatic cholangiocarcinoma with BO, the combined treatment significantly improved the overall mean survival time and mean stent patency period (6.8 vs. 3.4 months; *p *= 0.02; 13.2 ± 0.6 vs. 8.3 ± 0.5 months; *p* < 0.001) [27]. However, intra-RFA reportedly exhibits several limitations; in particular, it cannot ablate lesions outside the biliary tract or destroy a tumour mass completely [27, 28]. If other treatment measures are not implemented in time, the residual tumour will grow rapidly, eventually leading to stent stenosis.

Most BTCs are hypovascular tumours and visualisation of tumour staining and tumour vascularity is difficult. Therefore, the therapeutic efficacies of transarterial chemoembolisation and transarterial radioembolisation are largely limited in patients with BTCs, particularly patients with extrahepatic cholangiocarcinoma. Continuous low-dose infusion of drugs can achieve a high local drug concentration; moreover, the toxicity of the drugs can be greatly reduced by hepatic first-pass metabolism. In addition, the synergistic effect of oxaliplatin and 5-fluorouracil is more effective for killing tumour cells. Oxaliplatin can effectively inhibit tumour cell growth by inducing ribosome biogenesis stress [29], while continuous arterial infusion of 5-fluorouracil may effectively kill tumour cells that recover from the mitotic block produced by oxaliplatin as they progress into the S phase [30]. Furthermore, hepatic arterial infusion of oxaliplatin can induce high local concentration ratios between peripheral tissues and tumours [31]. Because HAIC is not restricted to a single session, repeated treatments and multiple sessions can be performed to increase tumour destruction and prolong OS. HAIC is an effective treatment for various malignancies, including BTCs [22, 32, 33]. A phase 2 study reported that overall response and disease control rates of 67.6% and 89.2%, respectively, could be achieved with the use of HAIC with oxaliplatin and 5-fluorouracil for pCCA [23]. Therefore, intra-RFA+HAIC can induce potent local anti-tumour effects.

In our study, the median OS in the combination group was 13.2 months, which was slightly longer than that in a phase 3 clinical study (11.7 months) where patients with Ad-BTCs received systemic chemotherapy with cisplatin and gemcitabine [34]. The median OS (13.4 months) in a recent randomised clinical trial of gemcitabine plus S-1 therapy for Ad-BTCs was similar to that in our study [35]. Notably, the proportion of patients with BO was 44.7% and 41% in the phase 3 study and randomised trial, respectively, while it was 100% in the present study. Therefore, the survival benefit of intra-RFA + HAIC may be noteworthy. The results of HAIC therapy alone or combined systemic therapies have been reported in a few studies till date, although the outcomes are inconsistent. The median survival time reported here is similar to that reported by Kasai et al (median 14.6 months) [21] but lower than that reported by Cercek et al (median 25.0 months) [22] and Konstantinidis et al (median 30.8 months) [36]. In this study, the patients did not receive systemic chemotherapy and exhibited concurrent BO, which may have resulted in the different prognosis. In addition, BO was caused by various types of tumours; therefore, an accurate comparison of effects between different studies is difficult. We also compared the results for our combination therapy with those for intra-RFA + stent placement in previous studies by Yang et al [27] and Inoue et al [14] and found that SPT and OS in our combination group were similar to those in the former study, while the median SPT was similar to that in the latter study (7.6 months). However, SPT in our combination group was obviously longer than that in a meta-analysis of intra-RFA + stent placement, where the pooled weighted mean difference in stent patency was only 1.7 months [16]. Therefore, our future study will focus on comparing the efficacy of stent placement + intra-RFA+HAIC with that of stent placement + intra-RFA for the treatment of Ad-BTCs with BO.

The absence of metastatic diseases, a stricture of < 4 cm, and the level of BO in Bismuth types I–II were independent predictors of longer SPT and OS, probably because the ablation extent and heat penetration depth are limited in patients with larger tumour burdens and complicated anatomies. For example, in pCCA cases, intra-RFA may not achieve complete coagulation necrosis in intraluminal masses in the hilar region [37]. This limitation can be overcome by step-by-step ablation in cases with stenoses longer than 2 cm; however, complete ablation remains impossible. The higher the residual tumour burden after intra-RFA, the harder it is to control by compensating HAIC. From the perspective of biological tumour behaviour, the absence of distant metastasis may imply relatively slow growth of tumour cells and relatively less invasion [38]. In summary, these could be the reasons why the three factors were independent predictors of longer SPT and OS in this study. We also found that a history of previous treatments was associated with shorter SPT and OS. There are several reasons for this. First, the biliary structures were reconstructed by surgery, leading to a more complex anatomy and limiting the effects of intra-RFA [39]. Furthermore, chemoradiotherapy can change the biological tumour behaviour, leading to reduced sensitivity to the drugs used in HAIC. Because the therapeutic effect of intra-RFA+HAIC was restricted, SPT and OS were shorter in patients with a history of previous treatments than in those with no previous history of surgery, radiotherapy, or chemotherapy.

The common AEs caused by biliary tract operation procedures were similar to previously reported AEs, including pain, biliary infection, haemorrhage, and pancreatitis. The incidences and grades of AEs showed no significant differences between the two groups. Patients with serious biliary infection were cured with injections of potent antibiotics based on the findings of blood bacterial cultures and drug sensitivity tests. HAIC-related AEs can be divided into two categories, those caused by the arterial catheter and those caused by the toxicity of oxaliplatin and fluorouracil. The incidence of AEs in the former category was extremely low, and timely diagnosis and effective treatment can avoid their aggravation. AEs in the second category were managed by treatment interruption, dose modification, or symptomatic treatments.

To the best of our knowledge, this study is the first to report the efficacy and safety of stent placement + intra-RFA+HAIC and the associated prognostic factors in patients with Ad-BTCs and BO. However, the study has several limitations. First, it was a retrospective study, which may have resulted in unexpected deviation during data extraction and analysis. In particular, treatment heterogeneity occurred because of disease variation, patient preferences, and economic situations. Second, it is difficult to distinguish between intra-RFA and HAIC in terms of their contribution to the longer OS and SPT. Third, because the metal stent interfered with radiological response evaluation, the local tumour control rate for intra-RFA and HAIC was not explored. Finally, SPT was not accurately evaluated for patients who were lost to follow-up or did not exhibit BO recurrence during their lifespan. In the future, well-controlled, prospective studies with larger samples should investigate the effect of these two novel therapeutic options on survival benefit and stent patency.

In conclusion, our findings suggest that stent placement combined with intra-RFA and HAIC is a feasible and safe option for the palliative treatment of Ad-BTCs with BO. Further prospective, multicentre, randomised controlled studies with more samples are necessary to further validate these findings.

## Abbreviations

Ad-BTCs: Advanced biliary tract cancers
AE: Adverse event
ALT: Alanine aminotransferase
BO: Biliary obstruction
BTCs: Biliary tract cancers
CI: Confidence interval
HAIC: Hepatic arterial infusion chemotherapy
intra-RFA: Intraluminal RFA
MBO: Malignant biliary obstruction
OS: Overall survival
pCCA: Perihilar cholangiocarcinoma
PTC: Percutaneous transhepatic cholangiography
PTCD: Percutaneous transhepatic cholangial drainage
RFA: Radiofrequency ablation
SAEs: Serious adverse events
SPT: Stent patency time
